# Physician beliefs and practice regarding end-of-life care in India

**DOI:** 10.4103/0972-5229.43679

**Published:** 2008

**Authors:** V. Theodore Barnett, V. K. Aurora

**Affiliations:** **From:** The John A. Burns School of Medicine, The University of Hawaii, Honolulu, Hawaii, USA; 1 LRS Institute of TB and Allied Diseases, New Delhi, India

**Keywords:** ICU, life-support, questionnaire

## Abstract

**Background and Aims:**

Physician beliefs and practices largely determine the withdrawal of life support in intensive care units. No information exists regarding beliefs regarding the withdrawal of life support among physicians in India.

**Materials and Methods:**

We performed a questionnaire at the NAPCON conference in Jaipur.

**Results:**

One hundred and twenty-two questionnaires were completed and returned. The majority of respondents did not apply do not resuscitate orders. Most physicians stated withdrawal of life support was not allowed or practiced at their institution. Thirty-five percent of physicians stated they performed life-support withdrawal. Barriers to good end-of-life care were primarily legal but also included hospital policy and social constraints.

**Conclusions:**

Pulmonary and critical care physicians in India have a lower rate of withdrawal of life support than western physicians. The reasons seem to be primarily legal and policy related. Culture and religion were not identified as barriers. Clarification of the legal and policy status of withdrawal of life support is needed

## Background

Physician beliefs regarding end-of-life care can have an impact on the care delivered to the dying patient. These beliefs both reflect and shape the environment surrounding dying in a hospital. Patient and family choices are framed, and in cases limited, by physician beliefs regarding what is appropriate, and where the law and social norms are not clear, what is allowed.

Studies in Europe have shown that physicians of different nationalities and religions have differing views and practices regarding life support[1–4] and these studies are supported by the limited studies on beliefs which exist outside of the west.[5,6] No studies exist detailing the beliefs of physicians in India. We therefore performed a questionnaire-based study of the beliefs and practices of end-of-life care among pulmonary and critical care physicians in India. It was our hypothesis that these beliefs would give insight into the reasons behind the state of life-support limitation and withdrawal in India.

## Materials and Methods

A questionnaire was developed by the investigators. The majority of questions were tailored to deal with both general and specific aspects of end-of-life care in India. For comparison with previous studies in the west, a sample of questions was taken from a questionnaire of intensive care physicians in Europe.[1] The questionnaire was tested for content and clarity with a small group of physicians at a meeting in Delhi. Minor changes for ease of completion were subsequently made. The study was approved by the Institutional Review Board at The Queen's Medical Center in Honolulu, Hawaii.

The questionnaire was administered at NAPCON, the joint Congress of the National College of Chest Physicians of India and the India Chest Society. The Congress was in Jaipur, India in November of 2002. The questionnaires were distributed at the registration desk and were also individually given to delegates as they entered sessions. Approximately 400 questionnaires were distributed (the exact number of questionnaires distributed is unknown due to wastage by those distributing).

## Results

One hundred twenty-two questionnaires were completed and returned giving an approximate completion rate of 30%. Not every questionnaire had all questions answered, therefore the number of responses for an individual question is occasionally less than the total number of questionnaires returned.

Survey respondent's gender was overwhelmingly male (91%). The large majority of respondents were Hindu (86%), with a small number of Muslim (3%), and Sikh (2%); the remainder were other or none. The age group distribution was: <30, 18 (15%); 30-39, 46 (38%); 40-49, 34 (28%); 50-59, 11 (9%); 60-69, 6 (5%); >69, 7 (6%).

Physicians were asked to identify their medical specialty. 71 (58%) identified themselves as pulmonologists only, 24 (20%) listed Internal Medicine as their only specialty, 25 physicians (20%) included critical care as a specialty.

When asked “Do you currently apply do not resuscitate (DNR) orders in the event of cardiac arrest?”, 41% (50 of 122) of physicians responded that they applied either oral or written DNR orders, 25% (30/122) chose “No, these orders would limit the level of care to these patients”, and 34% (42/122) chose “One should attempt to resuscitate every patient in the Intensive care unit (ICU).”

When questioned regarding with whom do not resuscitate (DNR) orders are discussed, 5% (5 of 104) chose the patient, 92% (96/104) chose the family and 3% (3/104) chose both answers. In a question on final authority; “In general, the ultimate decision should be made by…”, 2% chose the patient, 7% the family, 18% the physicians, and 73% chose “combined decision.”

On the question of whether withdrawal of life support was allowed at their hospital 64% (78 of 121) answered no and 35% (43/121) responded yes. When asked whether withdrawal of life support was practiced at their hospital 54% (66 of 122) said no and 46% (56/122) answered yes. When the questions are evaluated together, five physicians answered that withdrawal was allowed but was not practiced; 17 physicians stated withdrawal was not allowed but was practiced. Only 35% of physicians (42 of 121) stated that they withdraw life support.

When asked if culture or religion were important just 39% (44/113) answered yes while 61% (69 of 113) answered that religion or culture were not important in end-of-life decision makingWhen asked, “In patients with no real chance of recovering a meaningful life, do you sometimes…”, 56% (63 of 114) answered “withhold sophisticated therapy”, 30% (34/114) answered “withdraw sophisticated therapy”, 11% (12/114) answered both, 3% (3/114) choose “deliberately administer large doses of medication until death ensues.”, and 2% (2/114) chose both of the later choices (percentages do not add up to 100% due to rounding).

Participants responded regarding whether “un-declared limitation of care (i.e. slow codes)…occur at your hospital”; 35% (38 of 108) answered yes while 65% (70/108) answered no.

**Figure 1:**
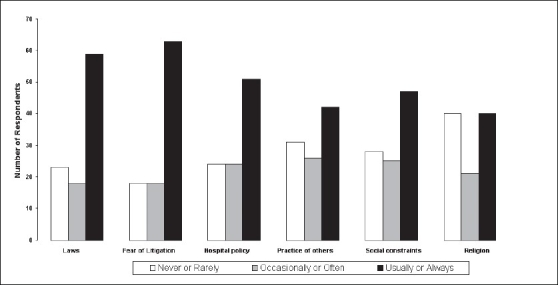
Importance of barriers to good end-of-life care as scored by questionnaire respondents

Respondents were asked to rate barriers to good end-of-life care at their hospital on a scale from 0 (not a barrier) to 5 (always a barrier). Responses are shown in Figure 1. For clarity, 0 and 1, 2 and 3, and 4 and 5, were combined. It can be seen that legal issues were ranked with the highest scores for barriers. Hospital policy and to a lesser extent social constraints and the practice of others were also seen as barriers. Religion was seen as a barrier by the fewest number of respondents.

**Figure 2:**
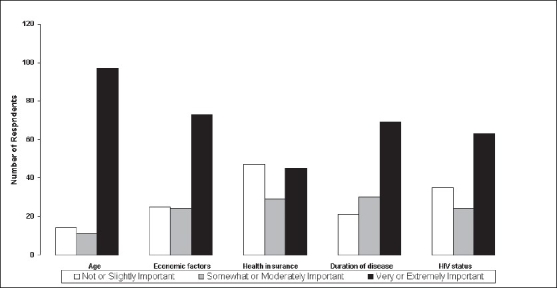
Perceived importance of patient factors in end-of-life decision-making

The importance of patient factors in making end-of-life decisions was also asked on a 0-5 scale. Zero was not important and 5 was extremely important. The results are grouped as above and shown in Figure 2. It can be seen that all the factors were considered very or extremely important except health insurance.

## Discussion

We found a minority of pulmonary and critical medicine physicians surveyed in India practiced withdrawal of life support. Survey participants worked at hospitals that by an almost two-thirds majority did not allow withdrawal of life support. Despite this, 46% answered that withdrawal of life support was practiced at their hospital indicating substantial withdrawal of life support despite perceived hospital policy.

Overall, the majority of physicians stated they did not apply DNR orders. When DNR orders were discussed, respondents, by an overwhelming majority, stated they discussed DNR orders primarily with the family. They endorsed a collaborative approach; almost three-fourths answered that the “ultimate decision” should be made as a combined decision between medical personnel and family.

The answers expressed by respondents regarding perceived barriers may help elucidate some of the reasons behind the low rate of limiting or withdrawing life support that was found. It is likely, but not certain, that the reasons for the low withdrawal rate in these hospitals are those expressed by survey respondents.

It has been stated that “the absence of guidelines for withdrawal and withholding of life support in Indian law is perceived to be the most important obstacle” to good end-of-life care.[7] Others have stated that withdrawal of life support is increasingly practiced.[8] Neither of these statements is referenced to supporting evidence. Our study finds the barriers to good end-of-life care in India as reported by physicians primarily were legal and administrative. There was uncertainty regarding institutional and legal standards. The limited end-of-life options in India were due to perceived bureaucratic but not ethical or cultural barriers. Recommendations have been made for the medical community to work to obtaining legislation that clarifies appropriate care.[9]

Most patient factors listed were considered important in end-of-life decisions. Of the choices presented, age was perceived as an important factor in decision-making by the largest percentage, followed by economic factors, duration of disease, and HIV status. All of these were considered important by greater than 60% of respondents. Only health insurance was chosen by less than 50%.

It has been argued that withdrawal of life support may be viewed differently by Hindus depending on the fulfilling of life ambitions[10] or “karmic thought” that may influence behaviors and perspectives on death and dying.[11] Outside India, Asian-Indian Hindu immigrants have a lower level of advance directives.[12] Culture and religion were not perceived to be barriers by a majority of survey participants. Religion ranked as the least important of the reasons given as a barrier. The above reviews of end-of-life beliefs in India suggest religion is a major consideration for patients and families.[11,12] However, when physicians were specifically asked, “Are cultural and religious differences important?” over 60% answered no. This may indicate a lack of influence of religion in decisions or an acceptance that it is an important factor but is not considered a barrier.

Published information on rates of withdrawal of life support in ICUs in India is limited and only available from two sources. A review of practice at ICUs in four hospitals in Mumbai showed limitation of care in only 34% of deaths and a very low (8%) incidence of withdrawal of life support.[13] A preliminary study from a hospital in New Delhi presented in a single table in a review article gives no details of the study or data collection but reports withdrawal of life-support, as it is generally defined in western studies, in no patients (the 4% of patients who had life support withdrawn and died in the hospital were brain-dead).[9] In this study patients who left against medical advice were counted as withdrawal of care (this consisted of 16% of patients). The author explained: “LAMA (Left against medical advice) is a situation peculiar to this part of the world. We have regarded these as withdrawal of treatment unilaterally by the patient's family. All of these were due to financial constraints.”[9] In a review of end-of-life care in India, Firth stated; “In India, the patient can be taken home, which implicitly discloses to the patient that death is imminent.”[10]

It is possible that some respondents in this survey felt LAMA was withdrawal of life support. This possibility has never been explored and was not included in our study which was developed by physicians in both India and the United States. Dying patients leaving the ICU and hospital and returning home due to financial constraints is believed to be rare in western countries, although data is unavailable. It appears this is a specific group of patients which likely has resonance in many countries. The extent of this occurrence is unknown but we agree with Mani and we believe it should be considered as a legitimate category in future studies of end-of-life care in many parts of the world.

Compared with a previous study in Europe we found marked differences in attitudes regarding end-of-life care among physicians in India. Only 6% of physicians in Europe did not apply DNR orders, 59% of physicians in India did not apply these orders. DNR orders were discussed with the patient in 26% of patients in Europe but only 5% of patients in India. When asked about patients with no reasonable chance of survival, 93% of European physicians and 56% of Indian physicians would sometimes withhold life support. Seventy seven percent of European physicians but only 30% of Indian physicians would withdraw therapy. Forty percent of European physicians and 3% of Indian physicians would give large doses of medication to hasten death.[1]

Surveys of practice in the United States show that withholding or withdrawal of life support precedes most deaths although the variation between institutions is large. The variation in the rate of withdrawal of life support was 0 to 79%.[3]

The rate of withdrawal of life support in India may eventually be similar to that seen in the west but this is by no means inevitable or desirable. This is also true of many other countries in the non-western world. Certainly factors perceived as barriers to good end-of-life care in India should be removed; however the level of withdrawal which would result is unknown. End-of-life decisions are inherently dependent on context and culture and each country and society must find its own level of appropriate withdrawal of life support. To expect every society to decide on the same level of withdrawal of life support would be a misunderstanding of social norms and practices and the constructs determining beliefs regarding the end of life. In all cases clarity of intent and understanding regarding end-of-life decisions and care between patients, families, and medical personnel is essential.

Since this study was conducted, a position statement has been published by the Indian Society of Critical Care Medicine. It states “If the patient or family consistently desires that life support be withdrawn, in situations in which the physician considers aggressive treatment nonbeneficial, the treating team is ethically bound to consider withdrawal within the limits of existing law.”[14] Although explicitly not giving legal advice this does supply an ethical framework for physicians to use.

The limitations of this study are those common to all questionnaires. There may be a bias in the physicians who complete and return the questionnaires. The beliefs identified are limited to those who were motivated and able to attend the conference. In addition, the small sample size limits generalization of the results to even the subset of physicians in India who care for patients utilizing the ICU but gives ideas of probable responses of physicians in India involved in end-of-life care in the ICU. For future research, it is likely the issues raised are valid even if the exact percentages may be subject to survey bias and sample size. Additional studies are needed to determine results with a larger sample and evaluate how a consensus evolves.

## Conclusion

Pulmonary and critical care physicians in India have a lower rate of life-support limitation than western physicians, particularly regarding decisions to withdraw life support. The questionnaire sample size was small and potentially non-representative but the reasons given are multiple and generally indicate inability to withdraw life support due to legal and hospital barriers. Knowledge of the beliefs and perceived barriers are important as attempts are made to enable patients and physicians to make end-of-life decisions. Clarification of the status (legal, ethical, administrative, and professional) of limitation and withdrawal of life support is needed to allow physicians to practice in keeping with their beliefs and the wishes of their patients and families. Several answers indicate that withdrawal of life support occurs despite hospital policy forbidding withdrawal.

We believe studies on end-of-life care throughout the world should include the category of patient removal from the ICU and hospital for terminal care in anticipation of death as unilateral withdrawal of life support. This category should be clearly defined. It may be extensively utilized in much of the world and has been ignored in the literature.

## APPENDIX 1

### QUESTIONAIRRE

1. Do you currently apply DNR orders in the event of cardiac arrest?

- Yes, written DNR orders.
- Yes, oral DNR orders.
- No. These orders would limit the level of care to these patients.
- No. One should attempt to resuscitate every patient in the ICU.

2. If DNR orders are used, are they, as a general rule discussed

- with the patient?
- with the family?

3. In general, the ultimate decision should be made by

- the patient
- the family
- The physicians

4. Is withdrawal of life support allowed at your hospital?     YES     NO

5. Is withdrawal of life support practiced?           YES     NO

6. Do you withdraw life support?              YES     NO

7. What percentage of deaths occur following the withdrawal of life support? ____ %

8. In patients with no real chance of recovering a meaningful life, do you sometimes

- withhold sophisticated therapy (i.e., not start mechanical ventilation, dialysis, etc)?
- withdraw sophisticated therapy (i.e., discontinue mechanical ventilation, dialysis, etc.)?
- deliberately administer large doses of medication (e.g., barbiturates or morphine) until death ensues?

9. A 40-year old man without previous disease has a cardiac arrest secondary to extended myocardial infarction. Six days later, he is still in profound coma with decerebrate movements. A neurologist agrees that the patient has no chance to recover. The patient breathes spontaneously via a T-piece. Select your attitude in each of the three following conditions.

- The patient has no family. _____
- The family insists on withhold and withdraw. _____
- The family insists that everything be done. _____

- Continue full support, including mechanical ventilation if required.
- Withhold additional therapy (including mechanical ventilation or antibiotics if required), but continue care.
- Discontinue treatment (including intravenous fluids, feeding) and allow the patient to die slowly (ensure minimal comfort medication if required).
- Administer sedation/morphine to allow the patient to die rapidly.

10. Does quiet (un-declared) limitation of care occur (i.e. Do slow code/sham codes (codes which are not run aggressively in order to be unsuccessful)) at your hospital?

11. Are cultural or religious differences important?     YES     NO

    If so, how?_______________

12. Please rate the following as barriers to good end-of-life care in your hospital.

**Table:**

	Not a barrier	Rarely	Occasionally	Often	Usually	Always
Laws	0	1	2	3	4	5
Fear of litigation	0	1	2	3	4	5
Hospital policy	0	1	2	3	4	5
Practice of others	0	1	2	3	4	5
Social constraints	0	1	2	3	4	5
Ethical concerns	0	1	2	3	4	5
Religious customs	0	1	2	3	4	5
Other _____	0	1	2	3	4	5

13. How important are the following patient factors in making end-of-life decisions?

**Table:**

	Not important	Slightly	Somewhat	Moderately	Very	Extremely
Age	0	1	2	3	4	5
Economic factors	0	1	2	3	4	5
Health insurance	0	1	2	3	4	5
Duration of disease	0	1	2	3	4	5
HIV status	0	1	2	3	4	5

14. Are there facilitators of good end-of-life care at your institution, and if so what? __________

15. What are (or what do you consider) the characteristics of a “good death”? ____________________

16. Please add any additional comments regarding end-of-life care and death in the ICU. ____________________

**Table:**

**DEMOGRAPHICS**						
Age	<30	30-39	40-49	50-59	60-69	70+
Type of hospital						
Private						
University						
Public						
Other __________						
Specialty						
Internal Medicine						
Critical Care						
Pulmonary						
Anesthesiology						
Surgery						
Other __________						
Gender						
Male	Female					
Religion						
Catholic						
Hindu						
Muslim						
Protestant						
Sikh						
Other __________						
